# A Patient-Centered Digitally Enabled Smart Speculum for Cervical Cancer Screening in Cameroon: A Paired Comparative Clinical Study

**DOI:** 10.1016/j.mcpdig.2026.100402

**Published:** 2026-09-16

**Authors:** Karl Gwei Njuwa Fai, Tankou S. Conrad, Lobga Babila Galega, Tatah Fritz Mbu, Sam Yakam Yimga Blessing, Rodrigue Ntone, Yap Boum

**Affiliations:** a Homegrown Solutions for Health (HS4H), Yaoundé, Cameroon; b Global Innovation and Creativity Space (GICMED), Yaoundé, Cameroon; c Africa Centres for Disease Control and Prevention (Africa CDC), Addis Ababa, Ethiopia; d Medical Research Council (MRC) Gambia at London School of Hygiene and Tropical Medicine (LSHTM), Fajara, Gambia; e Faculty of Medicine and Biomedical Sciences, University of Yaoundé 1, Cameroon

## Abstract

**Objective:**

To determine whether a patient-centered, digitally enabled smart speculum reduces discomfort and improves cervical visualization compared with the traditional bivalve speculum during routine pelvic examination.

**Patients and Methods:**

In a paired comparative clinical evaluation, 81 women undergoing routine gynecologic examination in 5 facilities in Cameroon’s Centre Region, from June 1, 2024, to September 30, 2024, were examined with both a traditional and a smart speculum. The primary outcome was insertion discomfort on a 10-point visual analog scale; secondary outcomes were discomfort at opening and removal, percentage cervical visualization, and complete visualization of the transformation zone and external cervical os. Comparisons used the Sign test, Wilcoxon signed-rank test, and McNemar test (*P*<.05).

**Results:**

Discomfort was significantly lower with the smart speculum at every stage (*P*<.001 for insertion, opening, and removal). Percentage visualization clustered near 100% (Wilcoxon *P*<.001). Full visualization increased from 53 of 81 (65%) to 73 of 81 (90%) for the transformation zone and from 60 of 81 (74%) to 80 of 81 (99%) for the external cervical os (*P*<.001).

**Conclusion:**

A patient-centered, digitally enabled speculum substantially improves comfort and cervical visualization during pelvic examination. Locally manufactured and integrated into artificial intelligence–supported telemedicine workflows, it could reshape cervical cancer screening and reproductive health services in Africa and beyond. Within the Africa Health Security and Sovereignty agenda, it shows digitalization and local manufacturing converging: an instrument designed, built, and serviced on the continent, carrying its own imaging and working channel. Comparable devices in Colombia and Ghana, and automated visual evaluation now tested across 5 African countries, suggest a workable route to equitable, sovereign screening.

Cervical cancer is among the most preventable malignancies, yet it continues to cause disproportionate morbidity and mortality, with 94% of 350,000 global deaths in in 2022 reported from low- and middle-income settings, especially in Africa.^1^ The continent accounts for a substantial share, 23%, of global cervical cancer deaths, driven by low screening coverage, late presentation, and fragmented referral and treatment pathways.^2^ Treatment is where much of that fragmentation shows. Where onsite treatment exists at all, it is often limited to cryogen applied with a cotton-tipped applicator. Women who need loop excision or hysterectomy for invasive disease have to be sent to a tertiary center at a cost and a distance that many of them never manage. Single-visit see, screen, triage, and treat clinics using portable thermal ablation have narrowed that gap in several African settings, and a new screening tool is only useful if it fits inside a pathway of that kind instead of adding 1 more step to it.^3^ While the global health community has increasingly emphasized human papillomavirus vaccination and screening scale-up, the lived experience of women undergoing screening has received far less attention.

At the center of this experience lies the vaginal speculum, an instrument that has changed little since its introduction more than a century ago. For many women, the speculum is associated with pain, fear, loss of dignity, and vulnerability.^4^^,^^5^ In contexts where cultural sensitivities, gender norms, and mistrust of health services already pose barriers, negative experiences during pelvic examination can deter not only initial screening but also future engagement with reproductive health services.

Digital health has been promoted as a transformative force to address health inequities, improve access, and strengthen health systems.^6^ However, many digital health tools deployed in Africa are externally designed, vertically implemented, and poorly aligned with local realities.^7^ Too often, they prioritize data extraction or technological novelty over patient experience, provider workflows, and system integration.

The Africa Health Security and Sovereignty (AHSS) agenda reframes digitalization and local manufacturing as strategic pillars for sustainable health system transformation.^8^ Within this framework, health technologies are not merely clinical tools but instruments of autonomy, resilience, and equity. Devices that are patient-centered, digitally enabled, and locally produced offer an opportunity to bridge innovation with sovereignty.

Other specula and fibreoptic imaging devices already on the market make similar claims, and digital capture of the cervix is not proprietary. Any instrument that opens the vagina can be paired with a fibreoptic camera or with a smartphone at sufficient zoom, and several groups have shown this.^9^^,^^10^ Our comparison in this study was therefore not with those devices. It was with the instrument actually in daily use in the clinics we studied, the bivalve Cusco speculum, which is what a woman presenting for screening in these 5 facilities would otherwise have been examined with.

In this study, we evaluated a smart, digitally enabled vaginal speculum designed to improve patient comfort and cervical visualization while enabling digital capture of cervical images for artificial intelligence-assisted interpretation and telemedicine-based consultation. Our objectives were 2-fold: first, to assess its clinical performance compared with the traditional speculum; and second, to articulate its potential role as a scalable digital health platform aligned with the AHSS agenda for cervical cancer prevention and broader reproductive health services.

## Patients and Methods

### Study Design and Participants

We conducted a paired comparative study among adult women aged 21 to 49 years presenting for routine gynecologic examination at 5 health facilities in the Centre Region of Cameroon: CASS Nkoldongo; Clinic Espoire Mendong; Akonolinga District Hospital; Soa District Hospital; and Monatele District Hospital. Participants were consecutively recruited from June 1, 2024, through September 30, 2024, after providing written informed consent. Exclusion criteria included acute pelvic infection and any contraindications to speculum examination.

The study was approved by the Cameroon National Ethical Committee for Research on Human Health No. 2023/11/1604/CE/CNERSH/SP. In Cameroon, the National Ethics Committee for Research on Human Health is the body that authorizes research involving human participants, including research using investigational medical devices, and its approval is the clearance required before such a study can begin. Its review covered the device itself, the study procedures, and the participant information and consent documents. No separate clearance from another regulatory body is required for device research at this stage in Cameroon.

### Study Procedures

#### Randomization and Masking

Following enrollment, each participant underwent pelvic examination using both a standard vaginal speculum (Cusco type) and the investigational smart speculum. The order of device use was randomized at the individual level using site-specific computer-generated allocation sequences (R Statistical Software, sample package; R Foundation for Statistical Computing). Five independent randomization lists (1 per study site) were generated and applied sequentially based on participant order of recruitment.

After enrollment, baseline sociodemographic and obstetric characteristics were recorded. Participants were then positioned in the dorsal lithotomy position on a standard examination bed, and a privacy drape was used to minimize visual awareness of the device being used, thereby reducing expectation bias. For each examination, the assigned speculum was lubricated using a water-based lubricant and inserted according to standard clinical practice.

#### Discomfort Assessment

Discomfort was assessed at 3 predefined stages: immediately after insertion, before opening (insertion); after adequate expansion to visualize the cervix (opening); and removal.

At each stage, participants rated discomfort using a visual 0 (no pain) to 10 (worst imaginable pain) Universal Pain Assessment Tool, incorporating both numeric scoring and facial expression cues (Figure 1). Responses were recorded immediately to minimize recall bias.

**Figure 1:**
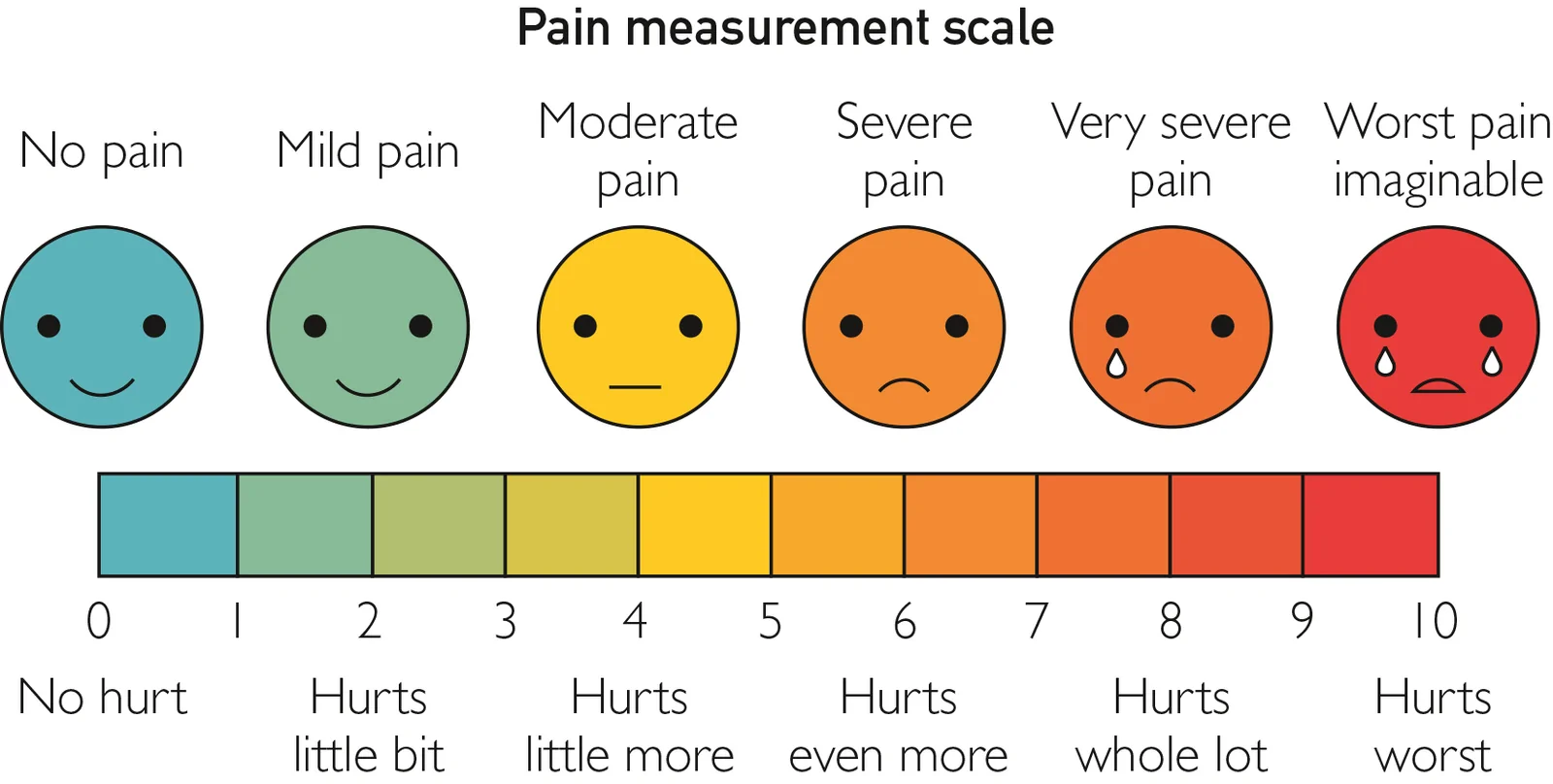
Universal Pain Assessment Tool used by participants to rate discomfort at each procedural stage, combining a 0 to 10 numeric scale with facial expression cues.

#### Cervical Visualization Assessment

Cervical visualization was assessed immediately after adequate exposure of the cervix. For examinations using the traditional speculum, the examiner directly visualized the cervix and recorded the visible area on a standardized schematic grid of the cervix divided into equal-sized squares (Figure 2A).

**Figure 2:**
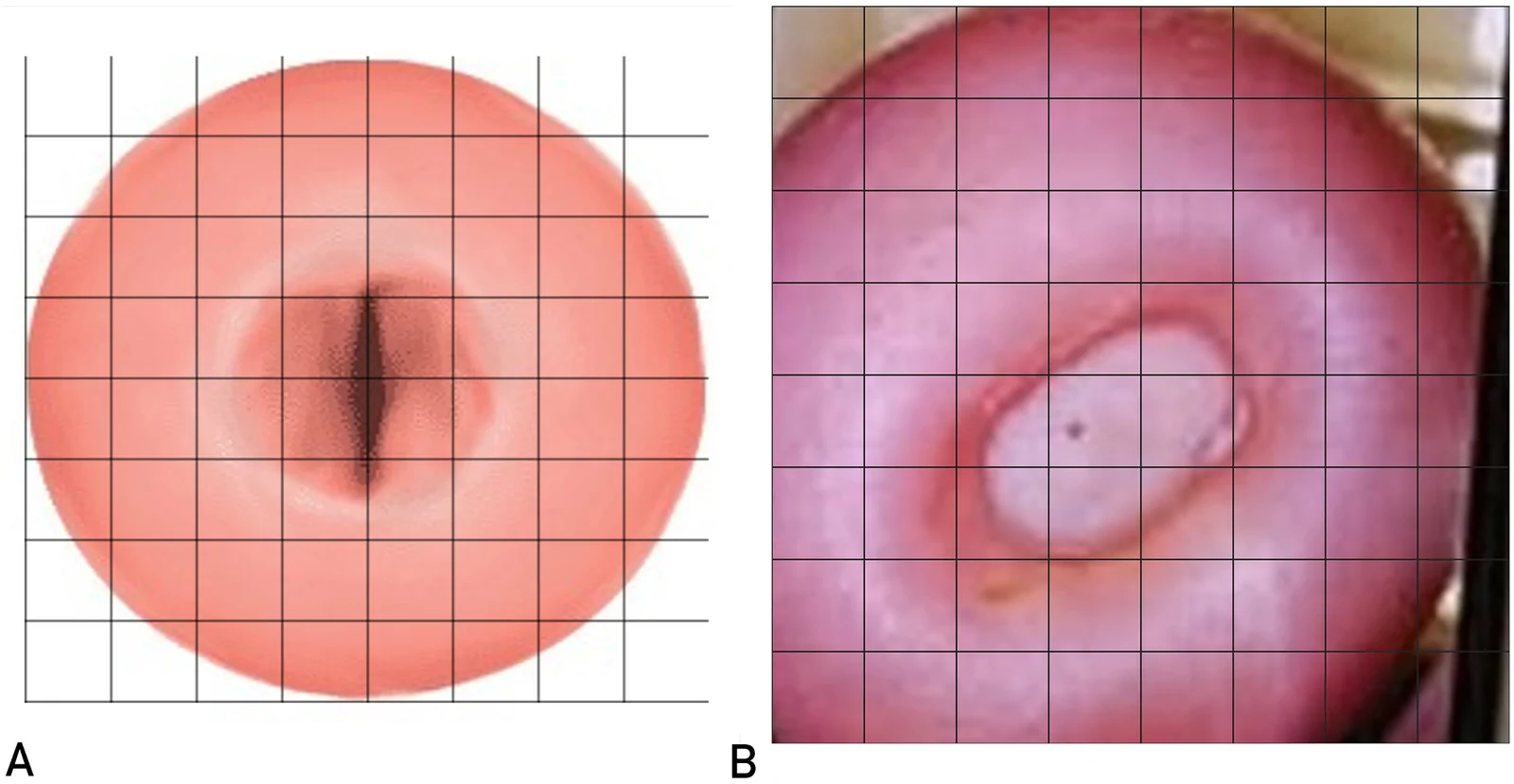
Standardized grid used to record the proportion of cervix visualized. (A) A schematic grid applied to a drawn cervix, used for examinations with the traditional speculum. (B) The same grid applied to a cervical image captured through the smart speculum during a study examination.

For examinations using the smart speculum, up to 3 cervical images were captured using the integrated imaging system. Images were reviewed immediately, and the visible cervical area was recorded by overlaying the same standardized grid on the captured image, so that the record for the smart speculum is a stored photograph of the cervix with the grid on it rather than a drawing (Figure 2B).

Two independent assessors, blinded to the type of speculum used, quantified the proportion of cervix visualized by counting shaded grid squares. The mean of the 2 measurements was used as the final estimate. Cervical visualization was calculated as follows:

Percentage cervical visualization = (number of squares shaded/total number of squares) × 100.

#### Device Description

The smart speculum is a digitally enabled vaginal speculum designed to improve patient comfort, optimize cervical visualization, and enable optional image capture (Figure 3). Smart speculum is the name under which the device was filed and patented, and it is used here as the name of a product rather than as a claim about the metal body on its own. What the name covers is the whole assembly, which is designed, built, and used as 1 instrument: the tubular body, the imaging channel with its camera and illumination, the separate working channel, and the paired mobile application. It is not a camera clipped onto a conventional speculum.

**Figure 3:**
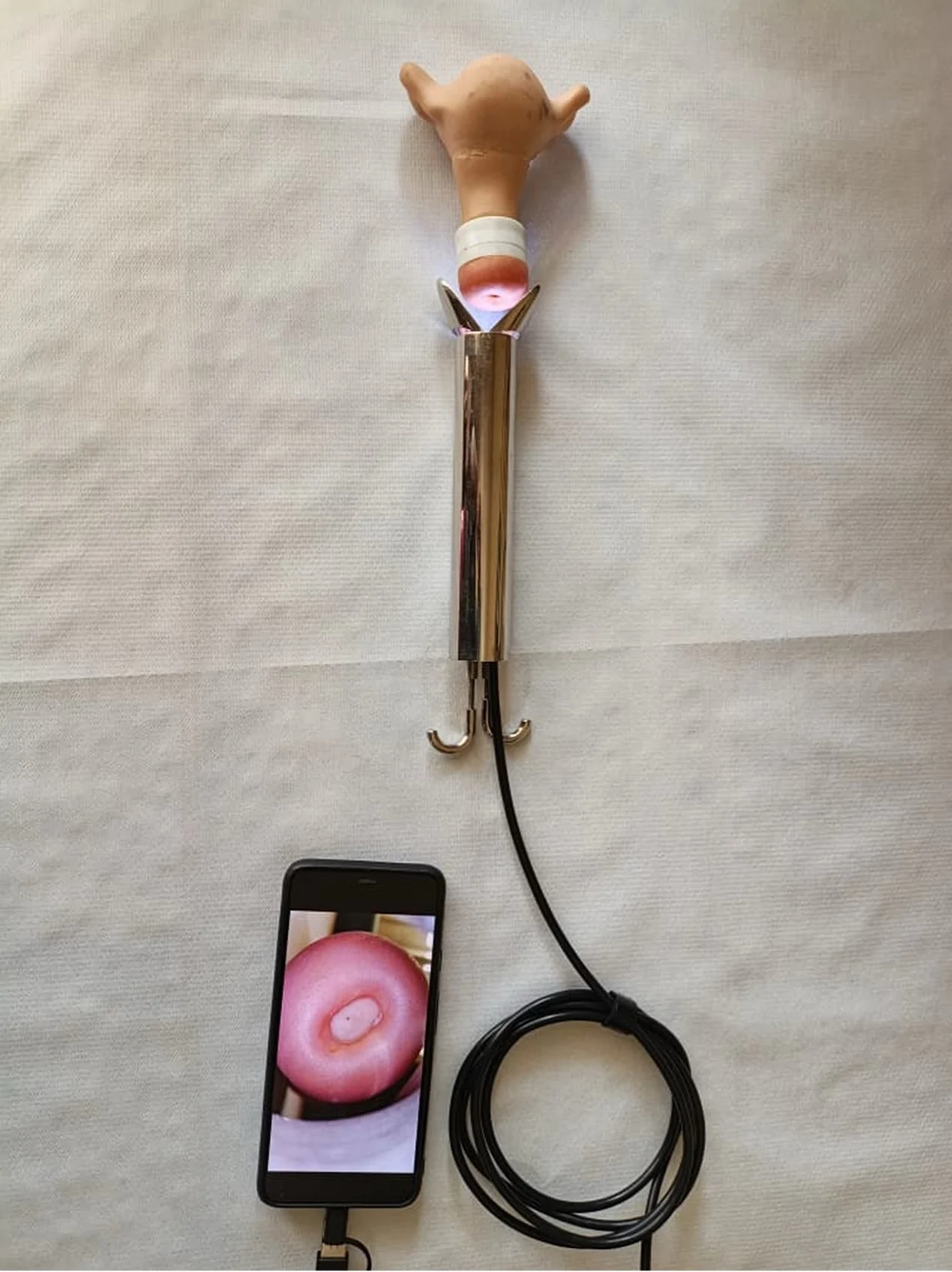
The smart speculum. The tubular body carries an imaging channel and a working channel; the endoscopic camera connects to a smartphone that provides power and display.

The device consists of an elongated cylindrical body (diameter, 29 mm) constructed from medical-grade stainless steel (AISI 304). The central body connects the anterior and posterior components and contains 2 internal channels: an imaging channel housing an endoscopic camera and a working channel allowing the introduction of ancillary instruments, such as swabs, biopsy forceps, or applicators of acetic acid.

The anterior component includes 2 semiconcave movable flaps attached via a mechanical linkage system. These flaps expand upward and outward during deployment, gently displacing the vaginal walls. When fully expanded, the flaps achieve a maximum separation of approximately 5.3 cm, enabling the visualization of the cervix with reduced lateral compared with the traditional speculum. A thin, single-use sheath made of medical-grade silicone is placed over the metal device before each use.

The imaging unit consists of a removable endoscopic camera with a 5-megapixel CMOS sensor, integrated LED Illumination, and flexible probe design. The camera connects to a smart device (eg, smartphone) via USB or android interface, which provides both power and display functionality. Light intensity can be adjusted via an inline control module.

The associated mobile application enables real-time visualization of the cervix; capture of still images and video; and secure storage of images locally or on a cloud-based platform. However, remote interpretation and artificial intelligence-assisted analysis were not evaluated in this study.

All components are detachable, enabling disassembly for cleaning and sterilization. The device is designed for reuse and can be sterilized using standard autoclave procedures and chemical sterilization methods.

### Outcomes

The primary outcome was participant-reported discomfort at speculum insertion. Secondary outcomes included discomfort at speculum opening or manipulation and removal, as well as the percentage of cervical visualization assessed. Binary secondary outcomes included complete versus incomplete visualization of the external cervical os.

### Statistical Analysis

Paired comparisons were conducted using the Sign test for discomfort scores; Wilcoxon signed-rank test for percentage visualization; and McNemar test for binary outcomes. Statistical significance was defined as *P*<.05. Analyses were conducted using R software.

## Results

### Sociodemographic Characteristics

A total of 81 women were included. The median age of participants was 33 years (range, 21-48 years), and the median body mass index (BMI) was 26.1 kg/m^2^ (interquartile range, 6.5 kg/m^2^) (Table 1). Regarding BMI classification, 1.2% were underweight, 37.0% had a normal BMI, 35.8% were overweight, and 26.0% were obese (Table 1). Parity ranged from 0 to 7 births (Table 1).

**Table 1:** Sociodemographic Characteristics of Included Participants

Characteristic	Value	
Age (y)	Median	33
	Range	21-48
Body mass index (kg/m^2^)	Median	26.1
	Range	18.3-50.8
	Interquartile range	6.5
	Underweight (<18.5)	1 (1.2)
	Normal (18-24.9)	30 (37.0)
	Overweight (25-29.9)	29 (35.8)
	Obese (>30)	21 (26)
No. of vaginal births	0	16 (19.8)
	1	17 (21.0)
	2	13 (16.0)
	3	12 (14.8)
	4	17 (21.0)
	5	4 (4.9)
	6	1 (1.2)
	7	1 (1.2)

Values are given as n (%) unless specified.

### Discomfort Level

Discomfort levels were compared between the traditional and smart speculums at 3 distinct stages: insertion, opening, and removal (Figure 4). The discomfort level on insertion was significantly lower with the smart speculum compared with the traditional speculum. The median pain score was substantially reduced, highlighting the enhanced comfort provided by the smart speculum. The statistical significance of this reduction was confirmed with *P* <.001 (Figure 4).

**Figure 4:**
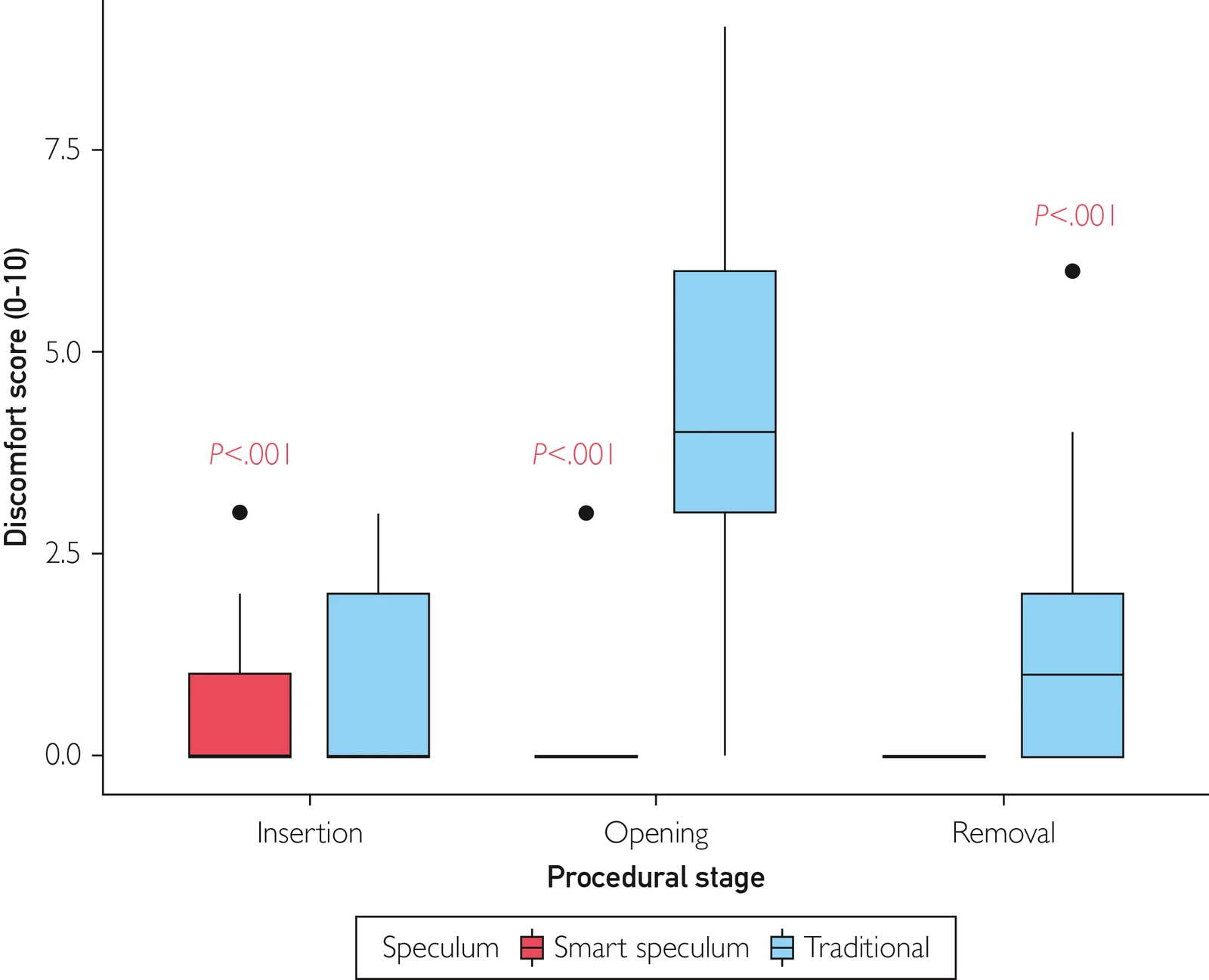
Participant-reported discomfort scores (0-10) by procedural stage and speculum type. Boxes show the median and interquartile range; whiskers extend to 1.5 times the interquartile range; points are values beyond that range.

The traditional speculum caused notably higher discomfort during the opening phase than the smart speculum. This phase exhibited the highest overall discomfort scores with the traditional speculum. The reduction in pain with the smart speculum was statistically significant (*P*<.001) (Figure 4).

The discomfort level at removal was also significantly lower with the smart speculum than with the traditional speculum. The difference in median scores again showed the improved comfort of the smart speculum, with *P*<.001 (Figure 4). Overall, discomfort was highest using the traditional speculum during the opening phase.

### Percentage Visualization

The percentage visibility of the cervix was compared between the traditional speculum and the smart speculum. The smart speculum showed a significant improvement in visibility compared with the traditional speculum (Figure 5). The smart speculum achieved nearly 100% visibility across most cases. The traditional speculum showed a broader range of visibility percentages, with a median of 80%, which was significantly lower than that of the smart speculum (Figure 5).

**Figure 5:**
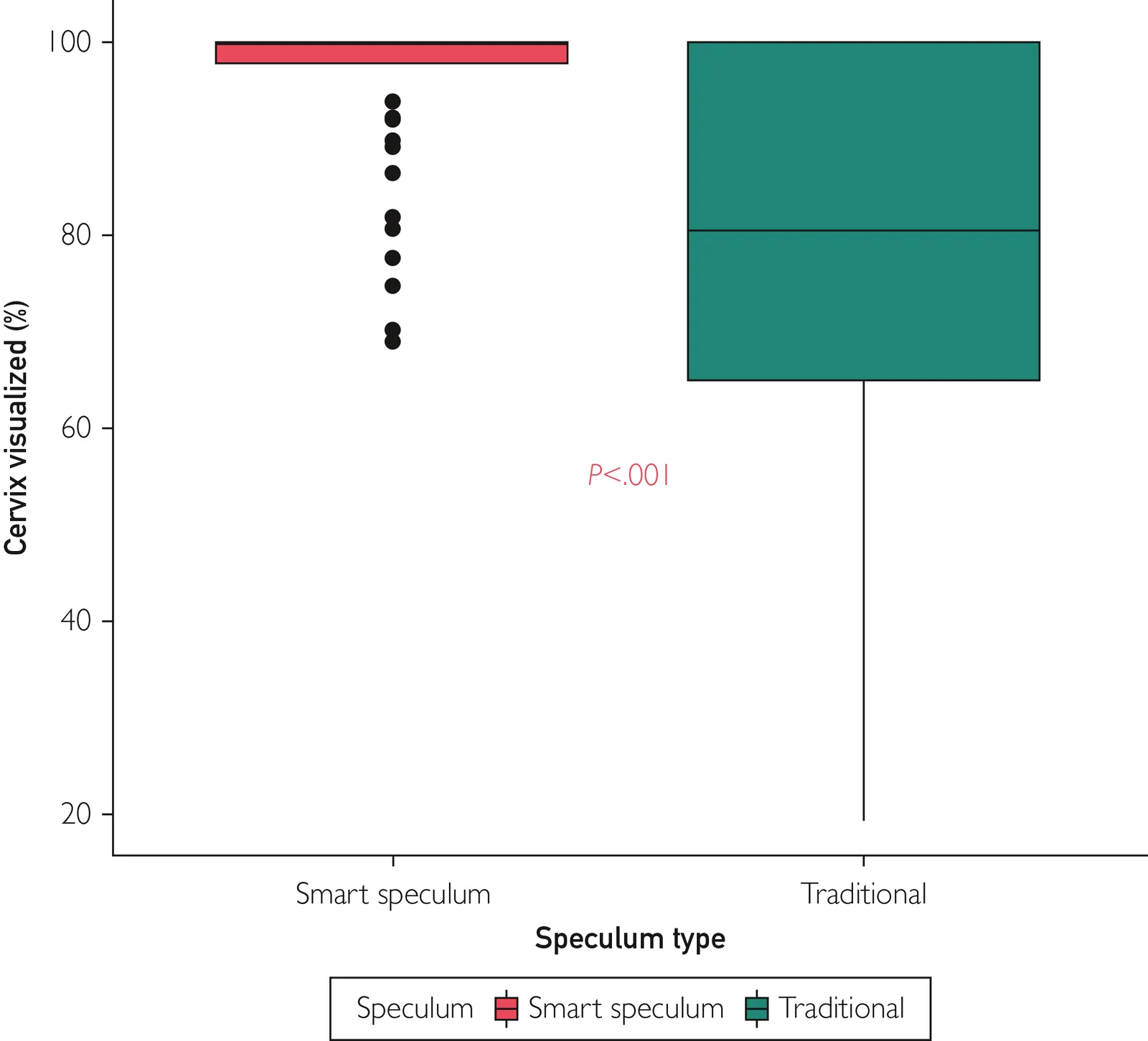
Percentage of the cervix visualized with each speculum. Boxes show the median and interquartile range; whiskers extend to 1.5 times the interquartile range; points are values beyond that range.

### Transformation Zone and External Os Visualization

Complete visualization of the transformation zone significantly increased from 53 of 81 (65%) for the traditional speculum to 73 of 81 (90%) for the smart speculum (Table 2). Complete visualization of the external cervical os significantly increased from 60 of 81 (74%) for the traditional speculum to 80 of 81 (99%) for the smart speculum (Table 2).

**Table 2:** Comparison of Visualization of the Transformation Zone and External Os Between the Traditional and Smart Speculums

Parameter	Traditional speculum	Smart speculum	*P*
Transformation zone			
Fully seen	53/81 (65%)	73/81 (90%)	<.001
Not fully seen	28/81 (35%)	08/81 (10%)	
External os			
Fully seen	60/81 (74%)	80/81 (99%)	<.001
Not fully seen	21/81 (26%)	01/81 (1%)	

## Discussion

This study demonstrates that redesigning a foundational gynecologic instrument through a patient-centered and digitally enabled approach can meaningfully improve both patient experience and clinical performance. The smart speculum was associated with substantially lower patient-reported discomfort compared with the traditional speculum across all procedural states, particularly during insertion and opening. At the same time, it markedly improved cervical visualization, with near-complete visualization in most cases and marked gains in visibility of the transformation zone and external cervical os. The results highlight 2 important but overlooked obstacles to successful cervical cancer screening: the actual experience of the examination^11^ and the technical quality of the visual display.^12^

A well-documented deterrent to screening uptake is the discomfort felt by patients during pelvic examination. Pain, embarrassment, and previous negative experiences have been found to lower both initial participant and adherence with subsequent screening in a range of settings.^13^ This barrier is especially prominent in low-resource settings, where structural barriers already restrict access to care. The reduction in discomfort observed with the redesigned speculum indicates that innovation at the device level may directly contribute to enhancing the acceptability of screening. Mechanistically, this likely reflects reduced lateral pressure on vaginal walls and more controlled expansion dynamics, which have been identified as key contributors to discomfort with the traditional speculum.^14^

The gains in cervical visualization carry clinical importance that goes beyond patient experience. Effective screening, especially using visual inspection with acetic acid and other visual-based diagnostic methods commonly employed in low- and middle-income countries,^15^ requires sufficient visualization of the transformation zone. Incomplete visualization may result in missed lesions, false reassurance, and reduced diagnostic sensitivity. The increase in complete transformation zone visualization from 65% with the traditional to 90% with the smart speculum represents a significant improvement in examination quality. Similarly, by almost completely visualizing the external cervical os, there is an improvement in the reliability of sampling and visual assessment.

These results are especially relevant given the global push toward eradicating cervical cancer. The World Health Organization strategy aims for 70% of women to be screened by age of 35 and 45 years,^16^ but in sub-Saharan Africa, many countries are still far below. Although much of the global response has been focused on scaling up human papillomavirus testing and vaccination, less attention has been paid to the point of care experience and tools used during examination. This study indicates that by making basic clinical instruments more usable and tolerable, we can add to current strategies by tackling the screening demand-related obstacles.

The integration of imaging capability within the device introduces additional potential, although it was not the focus of this evaluation. Capturing and reviewing cervical images at the point of care can aid documentation, quality assurance, and remote consultation/telemedicine. Previous studies have shown that digital cervicography and telemedicine methods can improve diagnostic consistency and broaden access to expert evaluation in settings with limited resources.^10^ Telemedicine enables remote expert review, referral decisions, and follow-up planning, particularly in rural and underserved areas. By reducing unnecessary referrals and enabling targeted escalation of care, telemedicine improves efficiency while preserving quality.^17^ However, further research is required to determine whether this integration of these capabilities into the smart speculum translates into measurable improvements in diagnostic accuracy.

More importantly, the local manufacturing of the smart speculum aligns directly with the AHSS pillar on strategic autonomy.^8^ Producing devices locally reduces dependency on imported technologies, shortens supply chains, lowers costs, and builds technical and industrial capacity. Importantly, it anchors innovation within African ecosystems, ensuring that design iterations respond to local needs and user feedback, which may enhance adoption and impact.^18^

In Colombia, CITOBOT was built through human-centered design and local prototyping as a portable screening device with an embedded classification model, meant to run in primary care rather than in referral hospitals.^19^ Across 5 African countries, automated visual evaluation of cervical images raised sensitivity for precancer from 36.6% with visual inspection alone to 60.1% and to 71.8% when the 2 were used together, which shows what image capture at the point of care can add once the images are good enough to work from.^20^ The Callascope, developed for low-resource settings and tested in Ghana, made the same argument from the hardware side by taking the speculum out of the examination altogether.^9^ What these have in common with our work is that the instrument, the software, and the setting were designed together and that production sits close to the people using it.

However, our study had several limitations. Although the paired design improved internal validity, the study was carried out in just 1 geographic area, which may restrict generalizability. Notwithstanding, the fundamental mechanisms underlying discomfort and cervical visualization are not expected to vary substantially across comparable clinical contexts, supporting the external relevance of the findings. Another limitation is that the discomfort was reported by the participants themselves and could have been shaped by personal perception or cultural influences. However, the paired-within participant design also represents a key strength, as it controlled for interindividual variability in pain perception, anatomy, and provider technique. Therefore, each participant served as their own control, increasing the sensitivity of the study to detect differences between devices, thereby strengthening internal validity. The way the view was recorded also differed between the 2 devices. With the traditional speculum, the examiner shaded the grid from what was seen directly at the bedside, while with the smart speculum, the grid was applied to a stored image (Figure 2B) that could be studied for as long as needed. We limited this by having 2 assessors, blinded to the device, score every record and by using the mean of their 2 scores, but the difference in how the view was captured remains a limitation, and a direct image-to-image comparison would settle it.

Future research using this device should focus on evaluating the impact of improved examination experience on screening uptake and adherence. Comparative studies evaluating diagnostic performance for detecting cervical lesions are also needed. Furthermore, implementation studies on integration into existing screening programs, cost-effectiveness, and acceptability among providers will be essential to inform scale-up.

## Conclusion

This study demonstrates that redesigning the vaginal speculum with the patient in mind can make pelvic examinations more comfortable and improve the view of the cervix. The findings indicate that improving the interface between patients and providers is a crucial factor in improving effectiveness and reach of cervical cancer screening programs, especially in settings with limited resources. This is an Africa-led solution to transform cervical cancer screening and reproductive health services, aligned with the AHSS agenda, benefiting Africa and offering lessons for global health in general.

## Potential Competing Interests

The authors report no competing interests.

## Ethics Statement

The study was approved by the Cameroon National Ethical Committee for Research on Human Health No. 2023/11/1604/CE/CNERSH/SP. In Cameroon, the National Ethics Committee for Research on Human Health is the body that authorizes research involving human participants, including research using investigational medical devices, and its approval is the clearance required before such a study can begin. Its review covered the device itself, the study procedures, and the participant information and consent documents. No separate clearance from another regulatory body is required for device research at this stage in Cameroon.
